# Amino Acid Derivatives of Ginsenoside AD-2 Induce HepG2 Cell Apoptosis by Affecting the Cytoskeleton

**DOI:** 10.3390/molecules28217400

**Published:** 2023-11-02

**Authors:** Lizhen Lin, Yuqing Zhao, Peng Wang, Tao Li, Yuhang Liang, Yu Chen, Xianyi Meng, Yudong Zhang, Guangyue Su

**Affiliations:** 1 School of Functional Food and Wine, Shenyang Pharmaceutical University, Shenyang 110016, China; 17852032780@163.com (L.L.); lyuhang0921@163.com (Y.L.); chenyyu2023@163.com (Y.C.); mengxianyi0813@163.com (X.M.); zyd1062969359@163.com (Y.Z.); 2 Key Laboratory of Natural Medicines of the Changbai Mountain, Ministry of Education, Yanbian University, Yanji 133002, China; 18841463340@163.com; 3 ORxes Therapeutics (Shanghai) Co., Ltd., Shanghai 200000, China; vipcfa@126.com; 4 Key Laboratory of Innovative Traditional Chinese Medicine for Major Chronic Diseases of Liaoning Province, Shenyang 110016, China

**Keywords:** AD-2, amino acid derivatives, apoptosis, cytotoxic, dammarane triterpene

## Abstract

**AD-2** (20(R)-dammarane-3β, 12β, 20, 25-tetrol, 25-OH-PPD) was structurally modified to introduce additional amino groups, which can better exert its anti-tumor effects in MCF-7, A549, LoVo, HCT-116, HT -29, and U-87 cell lines. We investigated the cellular activity of 15 different **AD-2** amino acid derivatives on HepG2 cells and the possible mechanism of action of the superior derivative **6b**. An MTT assay was used to detect the cytotoxicity of the derivatives. Western blotting was used to study the signaling pathways. Flow cytometry was used to detect cell apoptosis and ghost pen peptide staining was used to identify the changes in the cytoskeleton. The **AD-2** amino acid derivatives have a better cytotoxic effect on the HepG2 cells than **AD-2**, which may be achieved by promoting the apoptosis of HepG2 cells and influencing the cytoskeleton. The derivative **6b** shows obvious anti-HepG2 cells activity through affecting the expression of apoptotic proteins such as MDM2, P-p53, Bcl-2, Bax, Caspase 3, Cleaved Caspase 3, Caspase 8, and NSD2. According to the above findings, the amino acid derivatives of **AD-2** may be developed as HepG2 cytotoxic therapeutic drugs.

## 1. Introduction

Liver cancer is a global concern and a serious public health problem, and it is the fourth greatest cause of cancer-related mortality [1]. More than 90% of all cases of liver cancer are hepatocellular carcinoma (HCC) [2]. The main risk factors for HCC are hepatitis B, hepatitis C virus, metabolism, and non-alcoholic fatty liver disease [3,4,5]. Hepatic arterial chemotherapy is currently the main treatment method for liver cancer. Chemotherapy is frequently used to improve patient survival. However, chemotherapeutics are frequently linked with toxicity and severe side effects that compromise therapeutic efficacy and result in high death rates [6]. Natural products have the advantages of high efficiency and low toxicity and have a bright future. Therefore, it is necessary to develop natural products in anti-tumor drugs.

**Figure 1:**
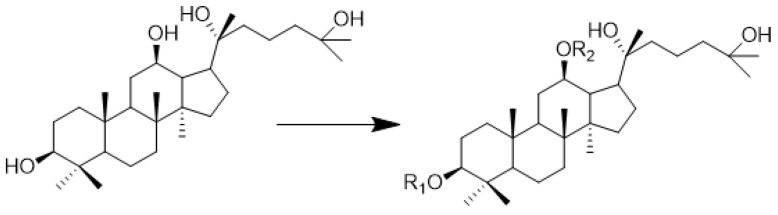
Chemical structure of **AD-2** and amino acid modification site.

Ginseng is a traditional Chinese medicinal herb known as the king of herbs. Ginsenosides have been proven to exhibit a variety of biological effects in pharmacological investigations, including anti-inflammation, anti-cancer, anti-diabetic disease, and cardiovascular protection [7,8]. 25-Hydroxylprotopanaxadiol-3β, 12β, 20-triol (**AD-2**) is a ginsenoside sapogenin with an anti-tumor effect obtained from the acid hydrolysis of ginseng (Figure 1). Previous studies have shown that **AD-2** has significant anti-lung cancer and anti-hepatic fibrosis activities in vitro and in vivo, and it is a potential anti-tumor lead molecule. However, its effect on liver cancer is weak. Amino acids are the basic unit of proteins, which have good biocompatibility and affinity. The modification with amino acids can improve the poor solubility and low permeability of the parent drug and its activity [9,10]. Therefore, amino acids are introduced into drug molecules to determine whether they enhance anti-tumor activity and to elucidate its mechanism of anti-tumor action (Figure 1 and Table 1).

Apoptosis is the most common form of cell death in physiological condition, and its main purpose is to remove damaged cells. The disorder of the apoptosis mechanism is related to the pathogenesis of cancer. Most clinical anticancer drug therapies exert their efficacy by inducing apoptosis [11]. Therefore, many efforts and studies have focused on identifying compounds that can reactivate apoptosis, to make use of this cell death process in the clinical treatment of cancer [12].

NSD2 (Nuclear receptor binding SET domain-protein 2), known as MMSET or WHSC1, is a member of the NSD histone methyltransferase family. As an important regulatory mechanism in epigenetics, histone methylation affects transcriptional regulation and chromatin remodeling [13]. The high expression of NSD2 has also been detected in a variety of malignant tumors. *p53* is a tumor suppressor gene, which plays a variety of roles in diseases and normal cell life [14]. As a tumor suppressor, the *p53* expression level is low and dynamic under physiological conditions. It is the key target of various pathways and functions in vivo. Specific transcription targets and roles in subcellular pathways are important for determining specific *p53* responses activated in cells [15]. It has been confirmed that *p53* can selectively induce the apoptosis of tumor cells [16]. MDM2 is an E3 ligase, which can carry out ubiquitination reactions against various substrates, and has a positive effect on the proliferation of tumor cells [17]. Among negative feedback regulation mechanisms of *p53*, the core mechanism is regulated by MDM2. MDM2 can inhibit the transcriptional activity and stability of *p53*. At the same time, MDM2 is a target gene of *p53*, and its expression level is regulated by *p53*. As we all know, the intrinsic apoptosis pathway is initiated by the activation of *p53*. *p53* can directly regulate the expression of a large number of Bcl-2 family proteins, which can be divided into two types of proteins: pro-apoptotic proteins such as Bax, and anti-apoptotic proteins including Bcl-2 [12,18,19].

As an oncogene, Bcl-2 can evidently inhibit cell apoptosis, and the induction of Bcl-2 expression inhibits cell apoptosis [20]. The Bax gene is the most important apoptotic gene in the human body and belongs to the Bcl-2 gene family. The encoded Bax protein can form a heterodimer with Bcl-2 and inhibit the protein expression of Bcl-2. Research showed that the ratio of Bax/Bcl-2 proteins is the key factor in determining the strength of the inhibitory effect on apoptosis [21]. The caspase family are key enzymes that cause apoptosis. Once the signal transduction pathway is activated, caspase is activated, followed by a cascade reaction of caspases. Then, the enzymes in the cell are activated, which can degrade important proteins in the cell, and finally lead to cells irreversibly going towards apoptosis [22].

Beta-actin (ACTB), a highly conserved cytoskeletal structural protein, has been recognized as a common housekeeping gene for many years and is used as a reference gene. However, increasing evidence suggests that ACTB is aberrantly expressed in a variety of cancers, thereby damaging the cytoskeleton and affecting tumor growth [23].

Our previous study performed MTT experiments on MCF-7, A549, LoVo, HCT-116, HT-29, and U-87 cell lines with 20 synthetic amino acid derivatives and discovered that the introduction of additional amino acid groups on **AD-2** could increase its tumor cell killing effect [24]. However, the therapeutic effects of drugs containing these amino acids on liver cancer have not been reported. The present study aims to investigate the toxic effect of **AD-2** amino acids derivatives on HepG2 cells and to explore the potential mechanism of action by detecting the influence on the cytoskeleton.

## 2. Results

### 2.1. Effect of ***AD-2*** Amino Acid Derivatives on the Proliferation of HepG2 Cells

To assess whether **AD-2** derivatives affect the growth and viability of HepG2 cells, cells were exposed to different concentrations (0, 10, 20, 40, and 80 μM) of **AD-2** derivatives, followed by an MTT assay at different time points to carry out testing. As shown in Table 1, the amino acid derivatives of **AD-2** reduced the cell viability of HepG2 cells in vitro, and its effect is significantly better than that of **AD-2**. The above results suggest that amino acid derivatives of **AD-2** could inhibit cell viability and increase cell death.

As can be seen from Table 1, the amino acid derivatives of **AD-2** have stronger inhibitory activities on the proliferation of HepG2 cells than **AD-2** and **5-FU**. Among them, the compounds **3a**, **4b**, **5b**, **6a**, and **6b** have IC_50_ values less than 10 μm on HepG2 cells, and their inhibitory intensity is five times that of **5-FU**. When **AD-2** is substituted with the same substituent, the substitution activity of C3 is slightly better than that of C12, which may be because the free -OH at C3 inhibits its activity (**4b**, **5b**, **6b**). For C3 substitution, the substituent contains conjugated groups such as a benzene ring, and the activity of the substituted compound is slightly better than that of the linear group (**6b**). The presence or absence of the Boc protection group has little effect on the potency of the compound, so the -COOH terminal of amino acids has little effect on the inhibitory activity of the compounds.

### 2.2. Effect of ***AD-2*** Derivative ***6b*** on Cytotoxicity of Normal Cells

**Figure 2:**
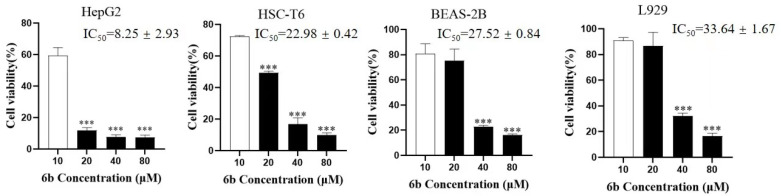
Toxicity determination of **AD-2** amino acid derivative **6b** (IC_50_ ± SD values, μM, 24 h) on cells of HepG2, HSC-T6, BEAS-2B, and L929. Data are expressed as means ± SDs of triplicate experiments performed independently (*** *p* < 0.001).

Through an MTT assay, we screened five compounds with good activity, namely **3a**, **4b**, **5b**, **6a**, and **6b**, the IC_50_ values of which are less than 10 μM. According to the experimental results, there is little difference in the efficacy of these five drugs, and the yield of **6b** is high in the process of drug preparation [25]. Considering the influence of solubility on the efficacy, derivative **6b** was selected for the following mechanism study. As shown in Figure 2, we tested the toxicity of derivative **6b** in the three normal cells HSC-T6, L929, and BEAS-2B at doses of 0, 10, 20, 40, and 80 μM, and found that the toxicity of derivative **6b** in these normal cells was significantly lower than that in HepG2 cells (*** *p* < 0.001).

### 2.3. Effect of Amino Acid Derivative ***6b*** of ***AD-2*** on Colony Formation of HepG2 Cells

**Figure 3:**
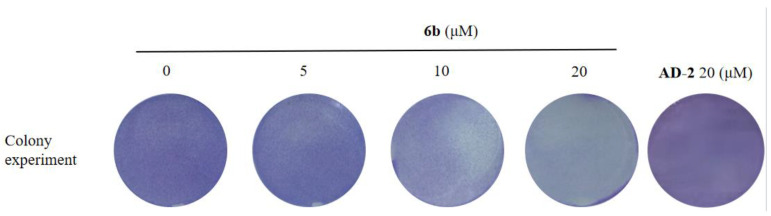
**AD-2** amino acid derivative **6b** inhibited the colony formation of HepG2 cells. HepG2 cells were treated with **6b** (0, 5, 10, 20 μM) for 14 days and stained with 0.1% crystal violet.

The colony formation method was used to detect the anti-proliferation and anti-survival ability of the amino acid derivative **6b** of **AD-2** (0, 10, 20, 40, and 80 μM) on HepG2 cells. The HepG2 cells were treated with different concentrations of **6b** for 14 days. As shown in Figure 3, **6b** inhibited the colony formation of HepG2 cells in a dose-dependent manner and almost eliminated the colony formation of HepG2 cells at 20 μM.

### 2.4. Effects of ***AD-2*** Amino Acid Derivative ***6b*** on Apoptosis of HepG2 Cells

**Figure 4:**
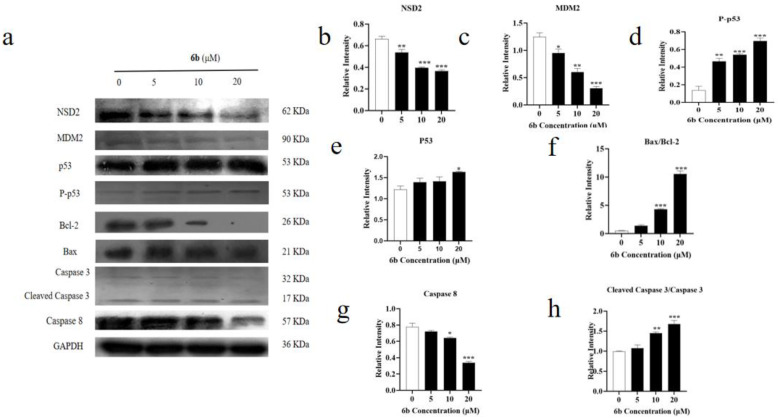
The expression levels of NSD2, MDM2, p53, P-p53, Bcl-2, Bax, Caspase 3, Cleaved Caspase 3, and Caspase 8 proteins were detected by Western blotting. (**a**) Protein band diagram; (**b**–**h**) protein quantification map. The data are expressed as the average standard deviation of three independent experiments. (* *p* < 0.05, ** *p* < 0.01, *** *p* < 0.001).

**Figure 5:**
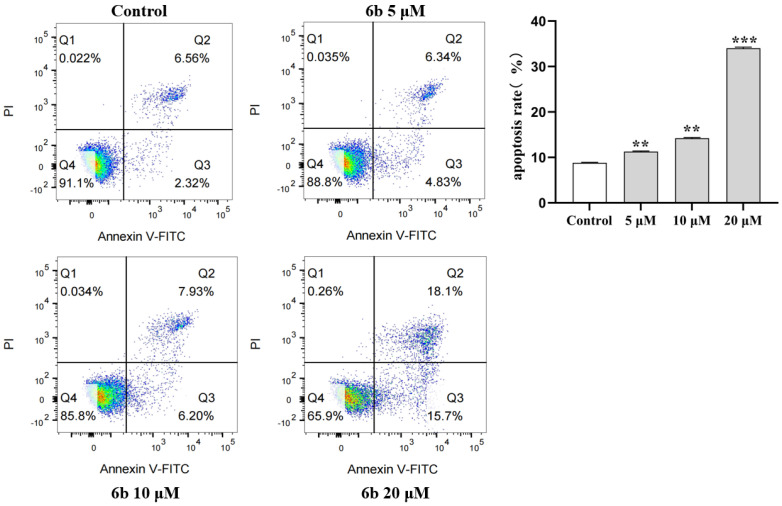
Detection of apoptosis of HepG2 cells induced by **6b** administration by flow cytometry. The cell apoptosis ratio is calculated based on the sum of the number of Q2 and Q3 phase cells in the figure. (** *p* < 0.01, *** *p* < 0.001).

The protein expression levels of NSD2, MDM2, P-p53, Bcl-2, Bax, Caspase 3, Cleaved Caspase 3, and Caspase 8 were detected in HepG2 cells treated with derivative **6b**. NSD2, a member of the NSD histone methyltransferase family, is highly expressed in a variety of malignant tumors. As shown in Figure 4a,b, the expression of NSD2 was reduced by the administration of derivative **6b**, indicating that **6b** may exert its cytotoxic effect on HepG2 cells by inhibiting the expression of oncogenes (** *p* < 0.01, *** *p* < 0.001). As shown in Figure 4a,c, the decrease in the expression level of MDM2 protein indicated that **6b** administration inhibited the proliferation of the HepG2 cells (* *p* < 0.05, ** *p* < 0.01, *** *p* < 0.001). As shown in Figure 4a,d, the increase in the P-p53 expression level after **6b** administration indicated that **6b** administration inhibited the proliferation of the HepG2 cells (** *p* < 0.01, *** *p* < 0.001). As shown in Figure 4a,e, the increase in the p53 expression level after administration of **6b** proved that **6b** promoted cell apoptosis (* *p* < 0.05). In the present study, **6b** increased the p53/MDM2, indicating that the mechanism of the proapoptotic effect of **6b** could possibly be related to the p53/MDM2 pathway. As shown in Figure 4a,f, Bcl-2 showed a decreasing trend after **6b** administration, and the ratio between the Bax/Bcl-2 proteins showed an increasing trend, indicating that **6b** possesses the effect of promoting apoptosis (*** *p* < 0.001). Furthermore, as shown in Figure 4a,g,h, compared to the control group, an increase in the Cleaved Caspase 3/Caspase 3 expression and a decrease in the Caspase 8 expression revealed that the administration could promote the apoptosis of the HepG2 cells by regulating the changes in the level of Caspase family proteins (** *p* < 0.01, *** *p* < 0.001). To further investigate the apoptosis of the HepG2 cells after **6b** administration, we used flow cytometry to detect their apoptosis. As shown in Figure 5, the results showed that compared with the control group, apoptosis (Q2+Q3) increased significantly with the increase in the **6b** administration concentration, and the percentage of apoptotic cells was 33.8% after 24 h of treatment with **6b** (20 μM).

### 2.5. Effect of Amino Acid Derivative ***6b*** of ***AD-2*** on Cytoskeleton

**Figure 6:**
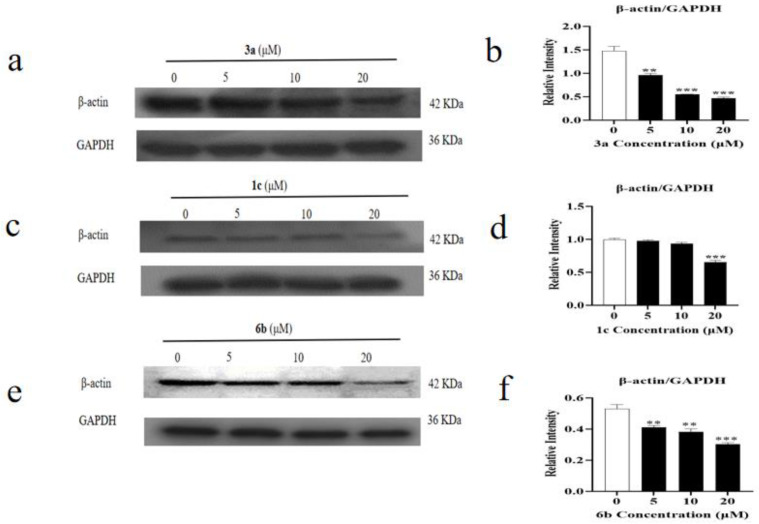
Mechanisms of derivative **6b** inducing the HepG2 cytoskeleton. (**a**–**f**) **3a**, **1c**, **6b** administration promotes the ablation of β-actin in HepG2 cells by Western blotting assay. Data are expressed as means ± SDs of triplicate experiments performed independently (** *p* < 0.01, *** *p* < 0.001).

**Figure 7:**
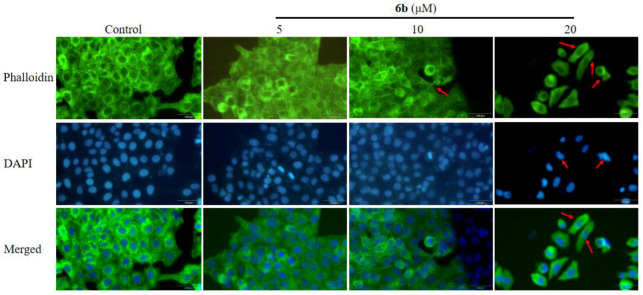
Phalloidin staining to detect derivative **6b** administration-induced ablation of HepG2 cytoskeleton. HepG2 cells were treated with **6b** (0, 5, 10, 20 μM) for 24 h. Actin was observed by phalloidin, and the nucleus was stained by DAPI. These images were captured with a fluorescence microscope at a scale of 100 μm, and the combined images of two kinds of staining were displayed. The arrow in the figure represents the place where morphological changes occur after administration.

Beta-actin (ACTB), a highly conserved cytoskeletal structural protein, has been recognized as a common housekeeping gene for many years and is used as a reference gene. However, there is growing evidence that ACTB is abnormally expressed in many malignancies, changing the cytoskeleton and influencing tumor formation. As shown in Figure 6, the decreased expression level of β-actin/GAPDH indicated that **6b** administration might affect the apoptosis of the HepG2 cells by affecting the cytoskeleton (** *p* < 0.01, *** *p* < 0.001). In order to determine whether the amino acid derivatives of **AD-2** have regular and accurate effects on cytoskeleton, we further selected another compound **3a** with a high toxicity to HepG2 cells and **1c**, which was found to be effective for colon cancer by our laboratory staff. As shown in Figure 6, the expression level of β-actin/GAPDH decreased after **3a** and **6b** administration, which indicated that these amino acid derivatives of **AD-2** may affect the cytoskeleton of HepG2 (** *p* < 0.01, *** *p* < 0.001). As shown in Figure 7, to further verify this speculation, we assessed the major structural changes in the HepG2 cell line after treatment with compound **6b** by staining the actin filaments and nuclei with phalloidin/FITC and DAPI, respectively. The qualitative results from treatment with 5, 10, and 20 μM showed that compound **6b** promoted nuclear and cytoplasmic changes. By staining the F-actin filaments, the exposure to different concentrations of compound **6b** resulted in the disruption of the cytoskeleton, and several stress bundles were observed, which were arranged in a disorganized manner. At the concentrations tested, one of the changes observed in nuclei by DAPI staining was DNA fragmentation. The fragmentation of genetic material is a characteristic of programmed cell death because apoptotic stimuli activate a cascade of caspases that cleaves and activates DNA fragmentation, which splits the DNA molecule into fragments of 180 base pairs. These results suggest that **6b** administration damages the cytoskeleton of HepG2 cells and promotes cell apoptosis.

## 3. Discussion

There are some issues with sorafenib as a first-line drug and multi-target inhibitor for cancer treatment, such as poor solubility, fast metabolism, and low bioavailability, which limit its further clinical application. Our research on amino acid derivatives is not as effective as clinical medication, but it is helpful to research and develop new drugs and has its own advantages [2,26,27]. Ginsenosides are the main active ingredient of ginseng and have a damarane-type basic skeleton. Studies on the structure–activity relationship of ginsenosides show that its anticancer activity is affected by the structure type of dammarane saponin, and the number and type of glycosyl units on the aglycone side chain also play a crucial role in anticancer activity [28,29]. The anticancer activity of ginsenoside has been widely reported. However, the effective methods of producing ginsenoside are limited, and it is difficult to meet the growing demand of human beings, especially for rare types. Due to the lack of ginsenoside, the correlation between its structure and anticancer activity has not been fully clarified [29]. Studies in the past decades have shown that amino acids play a major role in cancer metabolism and inhibits tumor growth [30,31]. Ginsenoside derivatives are a kind of derivative with promising cytotoxicity to HepG2 cells. This research group synthesized and characterized 28 new 25-OCH_3_ -PPD esters in the early stage, and found that they showed better anti-proliferation effects on A549 cells, Hela cells, HT-29 cells, MCF-7 cells, and IOSE144 cells [32]. Our previous research found that amino acid derivatives of AD-1 can inhibit cell proliferation and induce the apoptosis of activated hepatic stellate cells [33]. Xiao Shengnan et al. synthesized 27 kinds of ginsenoside piperazine derivatives and found that they had effects on PC-3 cells by inducing G1 phase arrest and apoptosis mediated by reactive oxygen species [34]. We modified the amino acid structure of **AD-2**, which helps develop new anti-tumor drugs, increased the further development and application of ginsenoside, and further studied the structure–activity relationship of ginsenoside against proliferation. **AD-2** is introduced with additional amino acid groups to make it polar, and protected amino groups to make it lipophilic. When esterase cleaves ester prodrugs in vivo, amino acid derivatives can also contribute to the solubility index of these compounds, so that they can still be decomposed into desired triterpenoids and harmless amino acids [25]. 

**Figure 8:**
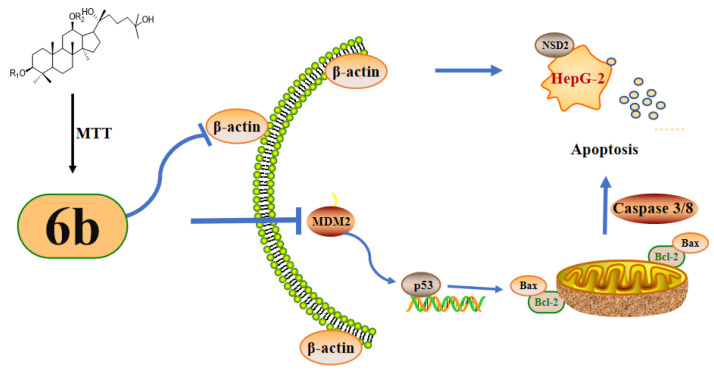
Proposed mechanism of compound **6b** for promoting apoptosis of HepG2 cells. To enhance the anti-hepatoma activity of **AD-2**, the amino acid derivatives of **AD-2** were introduced in the previous work. The amino acid derivative **6b** with high efficacy and low toxicity was screened by MTT assay. It has an excellent ability to resist the proliferation of hepatoma cell HepG2. The mechanism study found that **6b** could destroy the cytoskeleton, regulate the MDM2/p53 signaling pathway, increase the expression level of Bax/Bcl-2, and activate Caspase3/8 system, thus causing apoptosis, which was also verified by flow cytometry.

In the present study, we evaluated the cytotoxic activity of 15 amino acid derivatives of **AD-2** by using an MTT assay and found that their anti-toxicity to hepG2 cells was superior to **AD-2** itself. In addition, we investigated the cytotoxicity of the **AD-2** derivative **6b**, which has good HepG2 cytotoxicity, on three types of normal cells, HSC-T6, L929, and BEAS-2B. We found that their cytotoxicity to normal liver cells was weaker than that to HepG2 cells. Accordingly, we selected **6b** and carried out a Western blotting analysis to study the potential mechanism. As shown in Figure 8, the expression of NSD2, MDM2/p53, and Caspase 8 were lowered, while P-p53, Bax/Bcl-2, and Cleaved caspase 3/caspase 3 expressions were elevated to perform an anti-tumor effect. It was discovered that **6b** lowered NSD2, MDM2/p53, and caspase 8 expression while increasing P-p53, Bax/Bcl-2, and Cleaved caspase 3/caspase 3. These findings showed that **6b** may control apoptosis.

As we all know, cytoskeletons can lead to tumors by inducing cell proliferation and activating oncogenes [34]. The structural stability of cells mainly depends on the dynamics and function of the cytoskeleton, and actin filaments (also known as F-actin) are the main components of the cytoskeleton [35,36,37]. Therefore, phalloidin staining of F-actin is used to examine the cytoskeleton. After **6b** administration, the expression level of β-actin is decreased, indicating that the amino acid derivatives of **AD-2** may exert their apoptosis-promoting effects by affecting the cytoskeleton in HepG2 cells. The main structural changes of the HepG2 cell line treated with compound **6b** were evaluated by staining the actin filaments and nuclei with phalloidin/FITC and DAPI, respectively. The qualitative results of the treatment with 5, 10, and 20 μM showed that compound **6b** promoted the changes in the nucleus and cytoplasm. After **6b** administration, the nuclear structure was destroyed, and it was in an ablation state. Several stress bundles were observed in the cytoskeleton, which were arranged chaotically and the actin was slender [38]. These changes indicated that compound **6b** administration could affect the cytoskeleton.

## 4. Materials and Methods

### 4.1. Chemical and Reagents

The structure and absolute configuration of **AD-2**(**(20)R-AD-2**) and its amino acid derivatives have been previously determined and provided by Professor Zhao Yuqing’s laboratory at Yanbian University [25,39]. 5-Fluorouracil (**5-FU**, F6173) was purchased from McLean Reagent Co., Ltd. (Shanghai, China). Bax (WL64715), Bcl-2 (WL01556), MDM2 (WL01906), and Cleaved Caspase 3/Caspase 3 (WL02117) were purchased from Wanleibio (Shengyang, China). P-p53 (ELA008) was purchased from EnoGene (Nanjing, China). Caspase 8 (13423-L-AP) was purchased from Proteintech (Chicago, IL, USA). NSD2 (abs118224) was purchased from absin (Shanghai, China). β-actin (GB1101) was purchased from Servicebio (Wuhan, China). GAPDH (AP0063) was purchased from Bioworld (Beijing, China).

### 4.2. Cell Cultures

HepG2 cells (human hepatoma cell line), HSC-T6 (rat hepatic stellate cells), L929 (fibroblast), and BEAS-2B cells (human normal lung epithelial cells) were obtained from the research group of Professor Zhao Yuqing, Yanbian University. All hepatic cancer cell lines and rat normal cell lines were cultured in DMEM medium, containing 10% fetal bovine serum and 1% penicillin-streptomycin at 37 °C in a humid atmosphere (5% CO_2_–95% air).

### 4.3. Cell Viability Assay

Cell proliferation inhibition caused by **AD-2** derivatives was examined using MTT (3-(4,5-dimethylthiazol-2-yl)-2,5-diphenyl-2H-tetrazolium bromide) assay. Briefly, HepG2 cells (15,000 cells/well) were seeded into a 96-well microtiter plate in three replicates and allowed to adhere overnight (37 °C, 5% CO_2_). Thereafter, the cells were incubated (37 °C, 5% CO_2_) with a range of **AD-2** derivative concentrations (0, 10, 20, 40, and 80 μM) for 24 h. The cells were subsequently incubated with 10 μL MTT (5 mg/mL) for 4 h, and then the MTT salt solution was aspirated and DMSO (100 μL/well) was added and incubated for 15 min. The optical density was measured using an ELISA (800TS, BioTek Instruments, Winooski, VT, USA) at 490 nm. Three replicates were performed for each treatment [40].

### 4.4. Cytotoxicity Assay

Toxicity of a highly potent **AD-2** amino acid derivative **6b** to normal cells was examined using the MTT (3-(4,5-Dimethyl-2-thiazolyl)-2,5-diphenyl-2H-tetrazolium bromide) assay. The three normal cell lines HSC-T6, L929, and BEAS-2B were used to determine cytotoxicity in the same way as HepG2 cell viability.

### 4.5. Colony Formation Analysis

HepG2 cells were seeded in six-well plates with 500 cells per well. After an incubation period of 12 h, **6b** (0, 5, 10, and 20 μM) was added to the cells, respectively. The cells were cultured in a cell incubator for 14 days and the culture medium was changed every 3 days. Then, it was fixed with 4% paraformaldehyde for 15 min and dyed with 0.1% crystal violet for 30 min. After cleaning with PBS, photos were taken of the six-well plate [41]. 

### 4.6. Western Blotting Analysis

Western blotting was used to determine protein expression of MDM2, p53, P-p53, Bcl-2, Bax, Caspase 3, Cleaved Caspase 3, β-actin, and GAPDH. HepG2 cells were treated with **6b** (0, 5, 10, and 20 μM) for 24 h in the six-well plate. The supernatants in the six-well plate were removed, and the cells were washed three times in 0.1 M PBS. RIPA lysis buffer was used to lyse the cells. The cells were placed in a 4 °C refrigerator for 30 min before being mechanically lysed with a cell scraper. Cellular lysates were transferred to 1.5 mL micro-centrifuge tubes and centrifuged (12,000 rpm, 15 min, 4 °C) [42]. The crude protein extract supernatants were aspirated into new 1.5 mL micro-centrifuge tubes and maintained on ice. The bicinchoninic acid (BCA) assay was used to quantify the crude protein [43]. SDS-PAGE was performed in a 10% gel with an equal quantity of protein loaded per lane. Following electrophoresis, the resolved protein bands were transferred to a PVDF membrane and blocked for 1 h with 5% bovine serum albumin (BSA) in TBST buffer. Following blocking, they were incubated at 4 °C overnight with the indicated antibodies (1:1000–1:3000) [44]. The PVDF membrane was then washed with TBST containing 0.1% Tween-20 before being incubated for 1 h at room temperature with a 1:5000 dilution of horseradish peroxidase-conjugated secondary antibody [45]. Positive bands were visualized on an X-ray film using an enhanced chemiluminescence system.

### 4.7. Flow Cytometry

The human hepatoma cells HepG2 were collected and operated on according to the directions of the Annexin V-FITC kit after being treated with different doses of **6b** for 6 h, and early death of the cells was identified by flow cytometry. A duration of 24 h after transfection, the supernatant and washing liquid of each group of cells were collected, the cells were digested with 0.25% trypsin (without EDTA), centrifuged together with the above supernatant and culture medium, and were washed twice with PBS. Pre-prepared 1× Annexin V Binding solution was used to prepare a cell suspension with a final concentration of 1 × 106 cells/mL [46,47]. The apoptosis rate was measured by flow cytometry (FACSVerse, Becton, Dickinson and Company, Franklin Lakes, NJ, USA).

### 4.8. Phalloidin Staining

Cells were cultivated in six-well plates for 24 h to reach a density of 50% confluence. At 37 °C, the medium was aspirated and the cells were washed twice with pre-warmed 1× PBS (pH 7.4). Cells were fixed with 4% formaldehyde solution in PBS for 10 min at room temperature. At room temperature, cells were washed three times with PBS for 10 min each. The cells were dehydrated with acetone or permeabilized with 0.5% TionX-100 solution for five mins at room temperature [48]. Cells were washed three times with PBS for 10 min each at room temperature. A volume of 200 μL of the prepared FITC-labeled phalloidin working solution was taken, the cells were covered on the six-well plate, and incubated at room temperature for 30 min in the dark [49]. Cells in coverslipped six-well plates were washed three times with PBS for five mins each. Nuclei were counterstained with 200 μL DAPI solution for approximately 30 s, then washed with PBS. For fluorescence observation under a fluorescence microscope (IX73, Olympus Sales Service Co., Ltd., Beijing, China), FITC excitation/emission filters (Ex/Em = 496/516 nm) and DAPI excitation 1 emission filters (Ex/Em = 364/454 nm) were selected.

### 4.9. Statistical Analysis

Data were obtained by three repeated tests and expressed as mean ± standard deviation. The quantitative data was analyzed using GraphPad Prism 9.0. For multiple group comparisons, a one-way analysis of variance and Tukey’s test were utilized. A value of *p* < 0.05 was regarded as the statistical significance level.

## 5. Conclusions

In conclusion, we found that **AD-2** derivatives can inhibit the cell viability of HepG2. The mechanism may be related to influencing the cytoskeleton and promoting the apoptosis of cells. These findings suggest that amino acid derivatives of **AD-2** might be potential cytotoxic candidates for the treatment of hepatocellular carcinoma.

## Figures and Tables

**Table 1:** Antiproliferative activity (IC_50_ ± SD values, μM, 24 h) of 25-OH-PPD and synthetic derivatives substituted with amino acids in HepG2 cell lines treated with concentrations of 0, 10, 20, 40, and 80 μM.

Number	Compound	R_1_	R_2_	IC_50_
1	**1b**	Boc-Ala	H	13.44 ± 0.38
2	**1c**	Ala	H	26.84 ± 4.45
3	**2b**	Boc-Val	H	20.68 ± 2.36
4	**2c**	Val	H	13.22 ± 2.52
5	**3a**	H	Boc-Gly	7.05 ± 1.27
6	**3b**	Boc-Gly	H	17.09 ± 4.1
7	**3c**	Gly	H	11.47 ± 1.58
8	**4b**	Boc-Pro	H	6.97 ± 2.71
9	**4c**	Pro	H	15.50 ± 2.70
10	**5a**	H	Boc-Met	11.28 ± 1.99
11	**5b**	Boc-Met	H	9.79 ± 1.92
12	**5c**	Met	H	20.14 ± 4.01
13	**6a**	H	Boc-Phe	8.30 ± 3.86
14	**6b**	Boc-Phe	H	8.25 ± 2.93
15	**6c**	Phe	H	11.23 ± 1.36
16	**AD-2**	H	H	>100
17	**5-FU**	-	-	43.8 ± 1.93

## Data Availability

The original data of this paper are uploaded to the figshare database (https://figshare.com/account/items/22648129/edit, accessed on accessed on 18 April 2023). https://doi.org/10.6084/m9.figshare.22648129, accessed on 18 April 2023.

## Abbreviations

HCC—hepatocellular carcinoma, NAFLD—nonalcoholic fatty liver disease, **AD-2** 25-Hydroxylprotopanaxadiol-3β, 12β, 20-triol, ACTB Beta-actin. Amino acid derivatives of **AD-2** exert their antitumor effects by affecting the cytoskeleton in HepG2 cells.
